# Genome-Wide Identification and Analysis of the Aux/IAA Gene Family in *Panax ginseng*: Evidence for the Role of *PgIAA02* in Lateral Root Development

**DOI:** 10.3390/ijms25063470

**Published:** 2024-03-19

**Authors:** Yihan Wang, Qi Wang, Peng Di, Yingping Wang

**Affiliations:** State Local Joint Engineering Research Center of Ginseng Breeding and Application, Jilin Agricultural University, Changchun 130118, China; 20221860@mails.jlau.edu.cn (Y.W.); 20210964@mails.jlau.edu.cn (Q.W.)

**Keywords:** *Aux/IAA* gene family, *Panax ginseng*, evolution, expression pattern analysis, auxin-responsive genes

## Abstract

*Panax ginseng C. A. Meyer* (*Ginseng*) is one of the most used traditional Chinese herbal medicines, with its roots being used as the main common medicinal parts; its therapeutic potential has garnered significant attention. *AUXIN/INDOLE-3-ACETIC ACID* (*Aux/IAA*) is a family of early auxin-responsive genes capable of regulating root development in plants through the auxin signaling pathway. In the present study, 84 *Aux/IAA* genes were identified from the ginseng genome and their complexity and diversity were determined through their protein domains, phylogenetic relationships, gene structures, and cis-acting element predictions. Phylogenetic analyses classified PgIAA into six subgroups, with members in the same group showing greater sequence similarity. Analyses of interspecific collinearity suggest that segmental duplications likely drove the evolution of *PgIAA* genes, followed by purifying selection. An analysis of cis-regulatory elements suggested that *PgIAA* family genes may be involved in the regulation of plant hormones. RNA-seq data show that the expression pattern of *Aux/IAA* genes in *Ginseng* is tissue-specific, and *PgIAA02* and *PgIAA36* are specifically highly expressed in lateral, fibrous, and arm roots, suggesting their potential function in root development. The *PgIAA02* overexpression lines exhibited an inhibition of lateral root growth in *Ginseng*. In addition, yeast two-hybrid and subcellular localization experiments showed that PgIAA02 interacted with PgARF22/PgARF36 (ARF: auxin response factor) in the nucleus and participated in the biological process of root development. The above results lay the foundation for an in-depth study of Aux/IAA and provide preliminary information for further research on the role of the *Aux/IAA* gene family in the root development of *Ginseng*.

## 1. Introduction

Roots are one of the most important nutrient organs required for plant growth and development. Their functions are mainly to support the solid growth of the plant, absorb water and minerals from the soil, transport water and minerals to the stem, and store nutrients. Auxin was earlier described as the “root forming hormones of plants”, and there is a long-standing link between these small molecules and root development [1]. Indole-3-acetic acid (IAA) can play an important role in root development through biosynthesis, metabolism, transport, localization, signal transduction, and interaction with other hormones [2,3,4,5]. These responses typically require the involvement of the *Aux/IAA* family, the auxin response factor (ARF) family, the small molecule upregulated RNA (SAUR) for auxin, and the Gretchen Hagen3 (GH3) family of auxin response genes [6,7,8,9].

Aux/IAA proteins are short-lived nuclear proteins that mediate interactions with different proteins through four conserved domains (I, II, III, and IV), and these conserved motifs provide physical binding sites that regulate the auxin signaling pathway. Domain I contains the conserved “L × L × L” residue and interacts with the transcriptional co-repressor TOPLESS1 (TOP) [10]. Domain II is important for the stability of Aux/IAA. Domain II contains the SLiM degradation determinant, and the sequence variants shared by the degradation determinants directly affect the stability of the interaction of Aux/IAA with the transport inhibitor resistant1/auxin signaling F-box (TIR/AFB) co-receptors [11,12,13]. Domains III and IV together form the type I/II Phox and Bem1p (PB1) domains, which are able to form a dimer with the carboxy terminal dimerization domain (CTD) of ARF proteins, preventing the activation of ARF in the absence of auxin and affecting the expression of downstream genes through the regulation of the transcriptional activity of ARF. Once stimulated by high auxin signaling, Aux/IAA can be ubiquitinated by interacting with the transport inhibitor resistant1/auxin signaling F-box (TIR1/AFB) receptor and subsequently degraded by the 26S proteasome, and the released ARF regulates the expression of auxin-responsive genes, which is how Aux/IAA is involved in the regulation of plant development [6,7].

In recent years, increasing evidence for the involvement of Aux/IAA in root development has been found. For example, AtIAA16 and AtIAA20 are involved in primary root formation, while some other IAAs (e.g., *AtIAA3*, *AtIAA8*, and *AtIAA12*) are involved in lateral root formation via mutation or gain-of-function mutation [14,15,16]. Stable mutations in domain II of *Oryza sativa* OsIAA23 (Os06g39590) exhibit pleiotropic defects in root tissues, including the lateral root and crown root [17]. It has been shown that auxin regulates the initiation of root meristem organization through MP/ARF5-dependent transcriptional changes, a process that requires the involvement of the PB1 domain of BDL/IAA12 and the C-terminal domain of MP/ARF5 [18]. Tatematsu et al. (2004) [19] reported that mutations in conserved domain II of MSG2/IAA19 exhibit unique defects in lateral root formation, that the relatively specific defects observed in *msg2/iaa19* gain-of-function mutants are the same as those observed in *nph4/arf7* loss-of-function mutants, and that MSG2/IAA19 and NPH4/ARF7 may constitute a negative feedback loop that regulates differential growth responses to lateral root formation. To date, Aux/IAA has been identified and functionally characterized in a range of model plants, field crops, and cash crops [20,21,22].

*Panax ginseng* is a perennial herbaceous medicinal plant of *Araliaceae*, and its main medicinal part is the root, which is of high medicinal value and can be widely used in the fields of food, medical treatment, health care, and cosmetics. The *Aux/IAA* gene family is extensively involved in the regulation of root development, so the identification of these genes in *Ginseng* is essential for understanding the root development process in this medicinal plant. We used a genome-wide identification approach to identify 84 *Aux/IAA* from the genome of *Ginseng*. To elucidate the genetic and molecular characterization of these genes, phylogenetic relationships, gene structure, conserved motifs, cis-regulatory elements, and interspecific collinearity were analyzed. The expression pattern of the *Aux/IAA* gene in different tissues was analyzed, and the root development-related gene PgIAA02 was screened. The function of *PgIAA02* was preliminarily verified using qRT-PCR, the yeast two-hybrid (Y2H) technique, and transgenic plants. These results laid the foundation for an in-depth understanding of the function of Aux/IAA in *Ginseng* root development and provided a molecular basis for the study of resolving the growth hormone signaling pathway in *Ginseng*, which may provide a possibility for the cultivation and development of new ginseng varieties with high quality and high yield.

## 2. Results

### 2.1. Genome-Wide Identification of Ginseng Aux/IAA Family Genes

**Figure 1:**
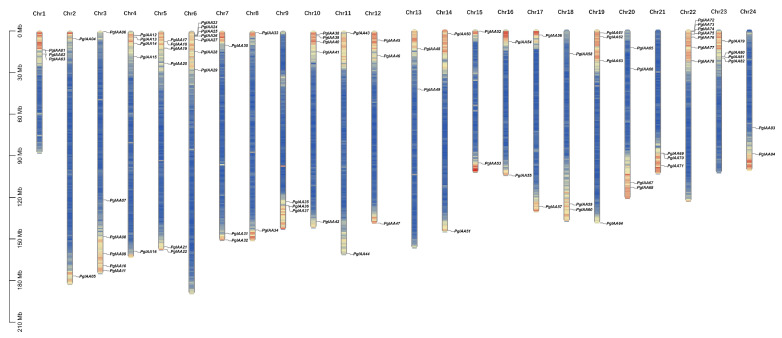
Distribution of gene density on 24 chromosomes of *Panax ginseng* and chromosomal localization of *PgIAA* family genes. The red region indicates a high density of gene number regions on the chromosome.

All *Aux/IAA* genes in *Ginseng* were searched via Aux/IAA conserved profiles from public ginseng genomic data resources using the HMMER search tool. Sequences that did not contain the PB1 structural domain were excluded from the SMART database and the NCBI conserved domain database, and 84 *Aux/IAA* genes were finally identified and renamed *PgIAA1-PgIAA84* based on their chromosomal locations (Supplementary Table S2). All 84 *PgIAA* genes were unevenly distributed on the 24 chromosomes and concentrated in the regions at the ends of the chromosomes. The most numerous genes were located on chr6 and chr22, and there was an average of two to five genes on other chromosomes (Figure 1). The 84 *PgIAA* genes encoded proteins of 156 (PgIAA82) to 1468 (PgIAA68) amino acids. By analyzing the physicochemical properties of the proteins, the molecular weight (MV) of the PgIAA proteins ranged from 17.41 to 162.31 and had an isoelectric point (pI) of 4.81–9.73 in between. The protein had a minimum instability index of 29.52 (PgIAA67) and a hydrophilicity grade between −0.715 (PgIAA15) and −0.17 (PgIAA66). Predictions of subcellular localization showed that the majority of the PgIAA proteins were localized in the nucleus, with the remaining family members localized to organelles such as chloroplasts, mitochondria, and the Golgi apparatus (Supplementary Table S2).

### 2.2. Phylogenetic Analysis of PgIAA Family Genes

**Figure 2:**
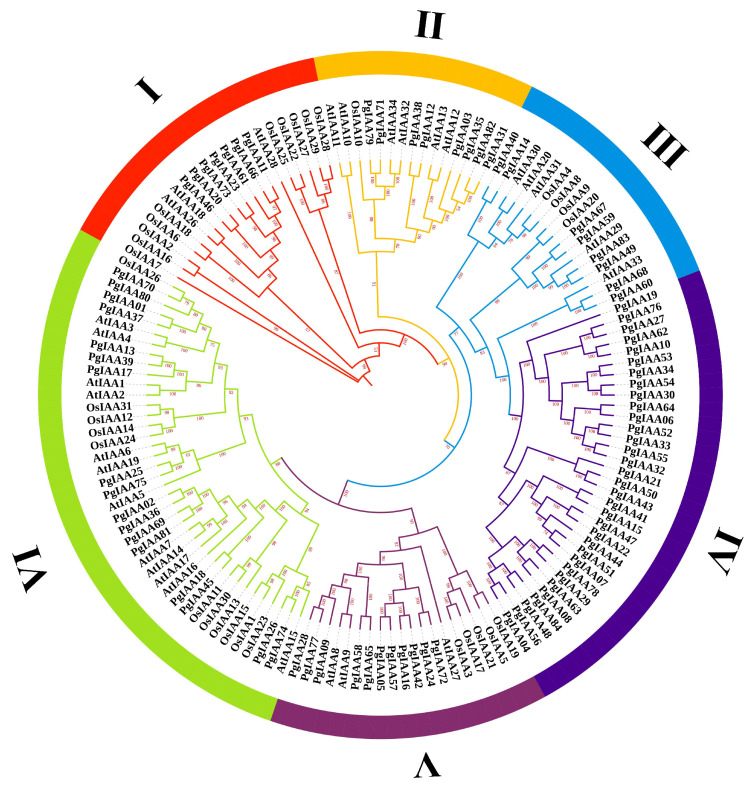
Phylogenetic tree of *Panax ginseng*, *Oryza sativa*, and *Arabidopsis thaliana* Aux/IAA proteins. A phylogenetic tree was built based on the JTTDCMut+F+R4 model using the maximum likelihood method IQ-TREE. I–VI are different subgroups.

There were 34 and 31 Aux/IAA members retrieved in *Arabidopsis thaliana* and *Oryza sativa*, respectively [20,23]. To further investigate the phylogenetic relationships between Aux/IAA proteins, we constructed a phylogenetic tree of all 149 Aux/IAA proteins (Supplementary Table S3), and the 149 proteins were classified into six groups (from I to VI) (Figure 2). As observed from the phylogenetic tree, in group IV, only the ginseng Aux/IAA members were included, indicating that the ginseng Aux/IAA family has a relatively conservative evolutionary relationship. In group V, PgIAAs were in the same branch as AtIAAs, whereas OsIAAs were clustered separately. *Ginseng* is more closely related to *Arabidopsis thaliana* than to *Oryza sativa*. Notably, in group VI, we found a set of sister pairs, PgIAA70 and OsIAA26, which are highly similar in sequence and are considered to be straight homologous genes that may have evolved through tandem and segmental duplication. Moreover, PgIAA02/36/69/81 clustered with AtIAA7/14/17 in *Arabidopsis*; the same Aux/IAA proteins are relatively highly conserved across species and may have similar functions.

### 2.3. Analysis of the Structure and Conserved Structural Domains of PgIAA Family Genes

To better understand the structural evolution of the *PgIAA* genes, we analyzed the structural composition of the genes of the Aux/IAA family members of *Ginseng* (Supplementary Table S4) using exons to show exon–intron structures. The number of exons ranged from 1 to 37 for all *PgIAA* genes, with the majority of genes containing 3–5 exons (52.3%) and *PgIAA68* containing the maximum number of exons, 37. The majority of the 84 *PgIAA* genes had two UTRs (46, 54.7%), 16 *PgIAA* genes did not contain UTRs (19.0%), and *PgIAA65* contained up to six UTRs (Supplementary Figure S1).

**Figure 3:**
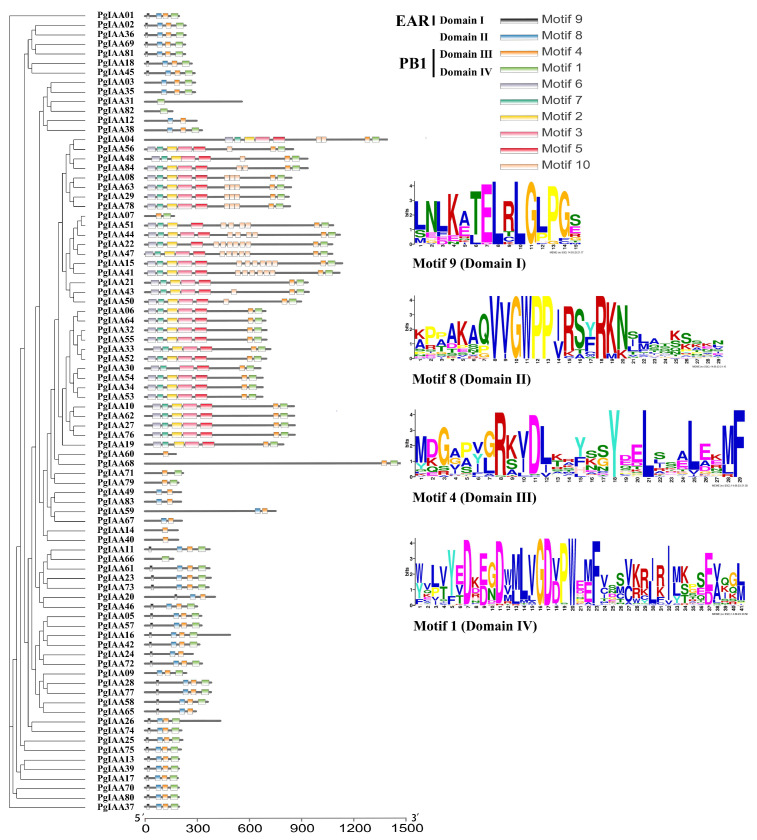
Conserved motifs and structural distribution of ginseng Aux/IAA proteins. Domain I is called the ethylene response factor (*ERF*)-associated amphiphilic repression (*EAR*) motif. The “GWPPV” motif in domain II is a degron, which controls the turnover of Aux/lAA proteins. Domain III and IV together form *type I/Ⅱ Phox* and *Bem1p* (*PB1*) domains.

The sequences of ginseng Aux/IAA family members were compared using the MAFFT program, and it was found that the protein sequences in the same subfamily had high similarity. Comparison results showed that 30 PgIAA proteins had a complete set of four conserved structural domains (domains I–IV) (Figure 3). The 33 PgIAA proteins contained the classical “L × L × L” structural domain, domain I, and the 42 PgIAA proteins contained structural domain II, which contains the protein motif “VGWPP”, associated with protein degradation and essential for protein stability (Figure 3). “VGWPP” is associated with protein degradation and is essential for protein stability, and 70 PgIAAs shared complete protein structural domains, domains III and IV (Figure 3). In addition to the four conserved structural domains of Aux/IAA proteins, six other conserved motifs were identified in PgIAA family proteins with an e-value ≤ 0.05. Approximately 32 Aux/IAA proteins, including PgIAA04, PgIAA06, PgIAA08, PgIAA10, and so forth, all contained Motif 2, Motif 3, Motif 5, Motif 6, and Motif 7, and 16 of these Aux/IAA proteins also contained Motif 10. Interestingly, none of the PgIAA proteins containing these six motifs contained conserved structural domain I and conserved structural domain II (Figure 3).

### 2.4. PgIAA Genes Cis-Acting Element Prediction

**Figure 4:**
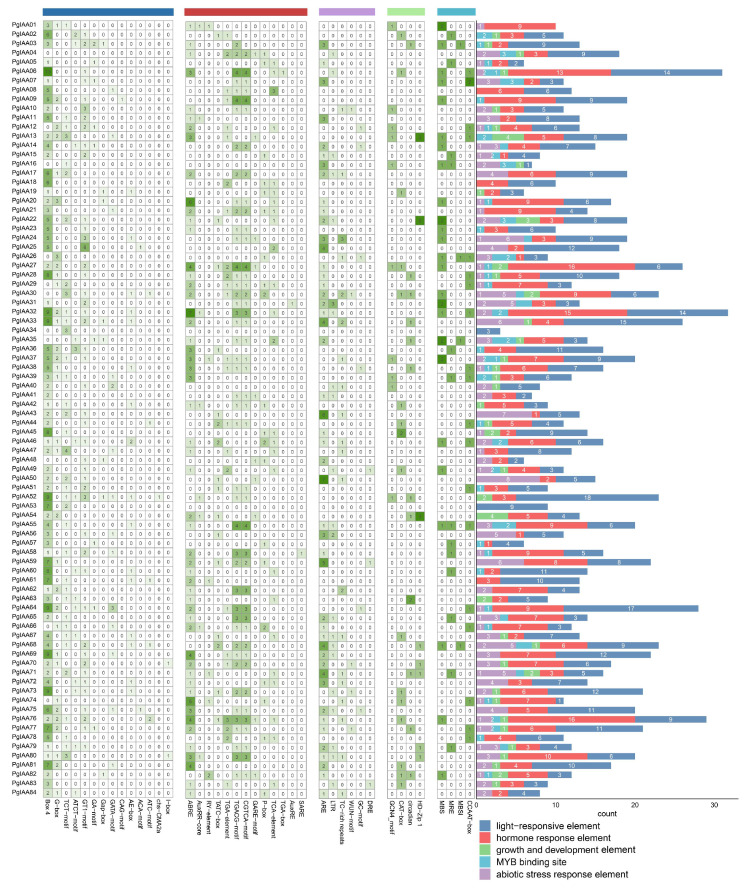
Predicted cis-regulatory elements of *PgIAA* genes. The heat map shows the number of cis-elements; the right side contains the species statistics.

The 1.5 kb upstream region of the *PgIAA* genes’ initiation transcription site was submitted to PlantCARE, and in total, 41 response elements were identified (Supplementary Table S5). These included 13 hormone response elements such as growth hormone response element (*AuxRE motif* and *AuxRR-core motif*), gibberellin response element (*P-box*), salicylic acid response element (*SARE*), methyl jasmone response element (*CGTCA-motif*), and abscisic acid response element (*ABRE*); *PgIAA27* and *PgIAA76* contain up to 16 hormone response elements (Figure 4). All PgIAA proteins contained light-responsive elements (*AAAC-motif*, *AE-box*, *Box II*, *G-box*, *GATA-motif*, *Cap-box*, etc.), and the *PgIAA64* promoter region contained the most light-responsive elements. There were 17 light-responsive elements. In addition, there were six abiotic stress-related elements, four growth and development elements, and four MYB-binding elements (Figure 4).

### 2.5. Duplication, Collinearity, and Evolutionary Analysis of the PgIAA Genes

**Figure 5:**
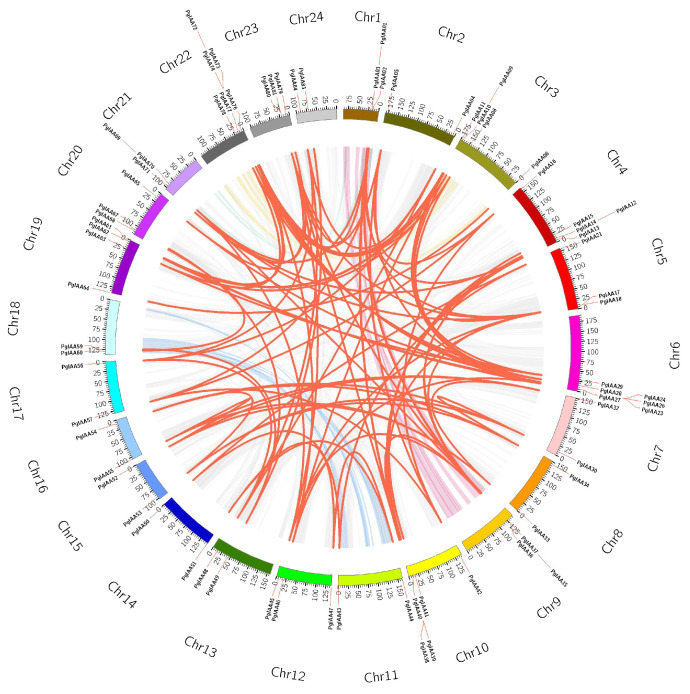
Chromosomal localization and collinearity analysis of the *PgIAA* genes. The gray lines in the background represent blocks of collinearity within *Panax ginseng* species. Red lines highlight *PgIAA* gene pairs with covariates.

Gene replication (tandem and segmental) and differentiation contribute to the expansion of gene families and the development of new functions. To study the evolutionary origin of *PgIAA* genes, intraspecific collinearity analysis was performed. The results showed that 52 pairs of *PgIAA* genes had covariance, with 2 pairs of *PgIAA* genes originating from tandem duplications and 50 pairs of *PgIAA* genes originating from segmental duplication events (Supplementary Table S6) (Figure 5). Gene replication usually causes gene mutations to gain new functions or divide ancestral gene functions that are essential for plant adaptation. In addition, the collinearity of *PgIAA* genes in *Panax ginseng* (*Pg*), *Panax quinquefolius* (*Pq*), *Panax stipulesnatus* (*Ps*), *Panax japonicus* (*Pj*), *Arabidopsis thaliana* (*At*), *Populus trichocarpa* (*Pt*), and *Daucus carota* (*Dc*) was analyzed (Supplementary Figure S2). The results showed that there were 5 pairs, 23 pairs, 18 pairs, and 45 pairs of *Aux/IAA* genes in collinearity between *Pg* and *At*, *Pt*, *Dc* and *Pq*, respectively. There were 9 pairs of *Aux/IAA* genes in collinearity between *Dc* and *Ps*, and 31 pairs of *Aux/IAA* genes in collinearity between *Pq* and *Pj*. The results of gene replication showed that the Ka/Ks ratio of the *PgIAA* gene was between 0.0666916 and 1.60068 (Supplementary Table S7). Among them, the Ka/Ks ratio of the *PgIAA25* and *PgIAA75*, and *PgIAA14* and *PgIAA40* gene pairs is greater than one. This indicates that the two pairs of genes have been positively selected in the complex evolutionary history, and that the Ka/Ks ratios of the remaining 49 pairs of *PgIAA* genes are less than one, indicating that most *PgIAA* genes are subjected to purification selection during evolution. These include *PgIAA02* and *PgIAA36/69/81*, so it is speculated that these genes have eliminated harmful mutation sites through purification selection during evolution.

### 2.6. Differential Expression Analysis of PgIAA Genes in Different Tissues and under Hormone Treatment

**Figure 6:**
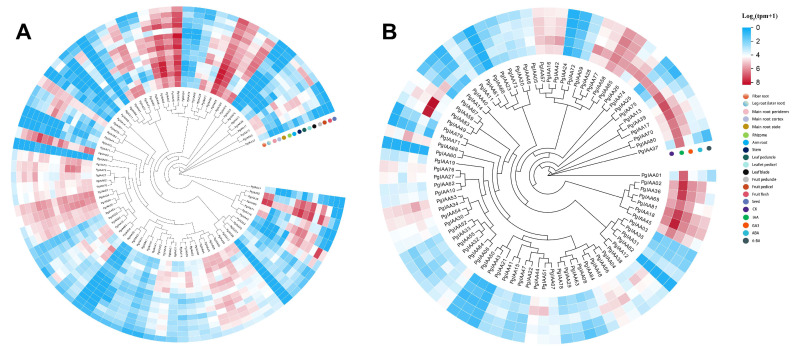
Gene expression pattern of *PgIAA* genes. (**A**) Expression levels of *PgIAA* genes in different tissues; (**B**) Expression levels of *PgIAA* genes under different hormone treatments. Gene expression levels were reflected as normalized TPM values in Log2 (TPM+1).

To explore the spatial and temporal expression patterns, the expression trends of *PgIAA* genes were analyzed in 15 different tissue sites, including fibrous roots, leg roots, main roots, stems, leaves, fruits, and seeds, with four hormone treatments using the public RNA-seq database, and a heat map of the expression of the *PgIAA* gene was generated (Figure 6) [24,25]. The results showed that *PgIAA42*, *PgIAA58*, and *PgIAA65* were highly expressed in 15 tissues. *PgIAA05*, *PgIAA16*, and *PgIAA57* were highly expressed in 14 sites except for their low expression in the leaf blade. *PgIAA02* and *PgIAA36* were highly expressed in fiber roots, leg roots, arm roots, stems, leaf peduncles, leaflet pedicels, and fruit peduncles (Figure 6A). The results of hormone treatment showed that *PgIAA02*, *PgIAA18*, *PgIAA36*, *PgIAA45*, *PgIAA49,* and *PgIAA83* could be highly expressed under IAA treatment. However, the expression level of the PgIAA gene was lower under other hormone treatments (Figure 6B).

**Figure 7:**
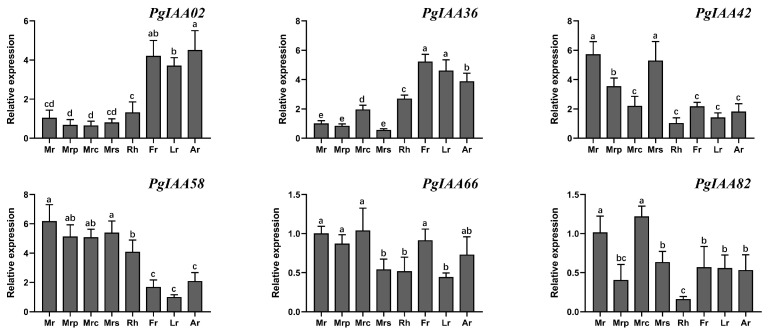
qRT-PCR analysis of tissue-specific expression of *PgIAA* genes. Relative mRNA expression levels of individual genes were normalized to the expression level of *β-actin*. The standard error of the mean of three replicates is shown. Letters above the error line mark the significance of the expression level. Main root (Mr), main root periderm (Mrp), main root cortex (Mrc), main root steles (Mrs), rhizome (Rh), leg root (Lr), fiber root (Fr), and arm root (Ar).

To validate the results of the RNA-seq expression analysis, quantitative real-time PCR (qRT-PCR) analysis was used to assess the expression of *PgIAA* genes in various tissues of *Ginseng* roots. This included the main root (Mr), main root periderm (Mrp), main root cortex (Mrc), main root steles (Mrs), rhizome (Rh), fiber root (Fr), leg root (Lr), and arm root (Ar) (Figure 7). The results showed that *PgIAA02* and *PgIAA36* exhibited organ-specific and high levels of expression in fiber roots, leg roots, and arm roots. *PgIAA42* and *PgIAA58* were highly expressed in the main root and various tissue parts of each main root, which might be related to the development of the primary root of *Ginseng*. *PgIAA66* and *PgIAA82* were genes with low expression in various parts of the root. The results of qRT-PCR analyses supported the expression patterns observed in the transcriptomic data, thus providing further evidence for the involvement of *Aux/IAA* genes in the growth and developmental response of *Ginseng*.

### 2.7. Subcellular Localization of PgIAA02/42/58 Proteins

**Figure 8:**
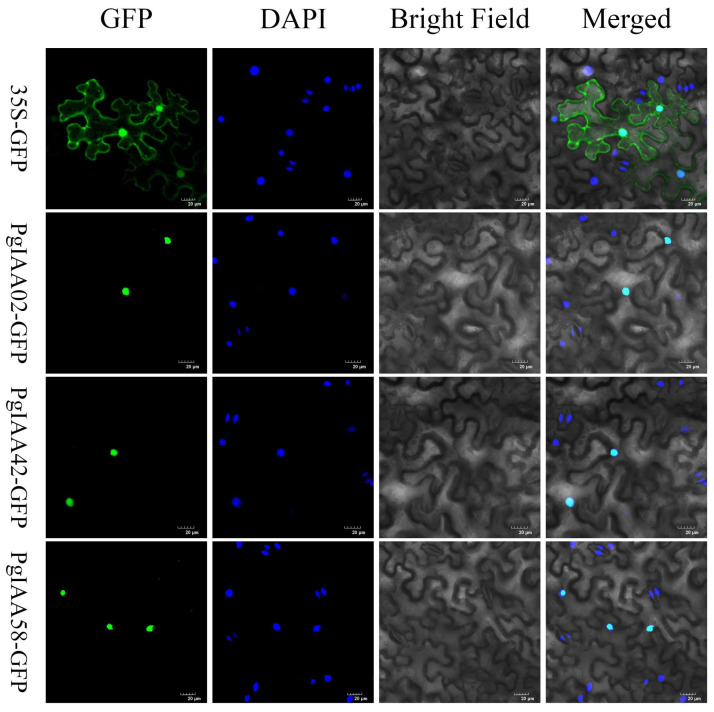
Subcellular localization analysis of PgIAA02/42/58 in *Nicotiana tabacum* leaves; 35S-GFP is the negative control, and the blue fluorescent signal of DAPI indicates the nucleus. Scale bar: 20 µm.

To further analyze the expression pattern of PgIAA proteins and their potential function in roots, *PgIAA02/42/58*, which is highly expressed in roots, was selected for subcellular localization analysis. The fusion expression vectors of 35S:PgIAA02-GFP, 35S:PgIAA42-GFP, and 35S:PgIAA58-GFP were constructed, in which the empty vector of 35S-GFP was used as a negative control, and the fluorescence signals of DAPI were used to indicate the nucleus. These vectors were transferred into tobacco leaves, and fluorescence was observed with a ×20 laser confocal microscope. The blank GFP signal was expressed in the membrane and nucleus, whereas the fluorescent signals of 35S:PgIAA02-GFP, 35S:PgIAA42-GFP, and 35S:PgIAA58-GFP were expressed predominantly in the nucleus (Figure 8), which was consistent with the results of predictive analysis (Table S2).

### 2.8. Overexpression of PgIAA02 in Arabidopsis thaliana

**Figure 9:**
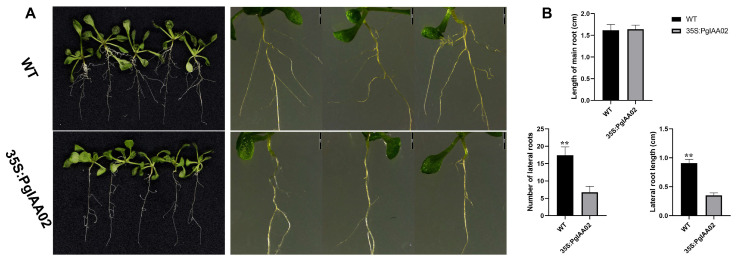
Wild type (WT) and 35S plants: PgIAA02 transgenic plant seedlings were grown on 1/2 MS medium for 10 days. (**A**) Growth phenotype. Scale bar = 2 mm. (**B**) Length of main root, number of lateral roots, and length of lateral roots. ** represents an extremely significant difference.

To predict the function of *PgIAA02* in root development, we constructed *PgIAA02* overexpression plants and further analyzed the root phenotype of transgenic plants (Figure 9). Wild-type (WT) seedlings were grown in 1/2 Murashige–Skoog (MS) medium for 10 days to observe the root phenotype. There was no significant difference in the main root length between the overexpression plants and the WT plants (Figure 9B). The expression level of PgIAA02 in the main root was low, which is consistent with the results of the prediction of the transcriptional expression profile (Figure 6A). Many lateral roots were produced on the primary roots of the WT plants, while the number of lateral roots of overexpression lines was significantly lower than that of the WT plans, and the length of lateral roots of overexpression plants was also lower than that of the WT plants, indicating that PgIAA02 changed the sensitivity of transgenic plants to auxin (Figure 9A). The number of lateral roots in the wild type was 61.32% higher than that in the overexpression plants. The average lateral root length of the overexpression plants was 0.91 cm, while the average lateral root length of the wild type was only 0.35 cm (Figure 9B). These results indicated that overexpression of PgIAA02 inhibited the growth and development of lateral roots.

### 2.9. The Interaction between PgIAA02 and PgARF22/36

**Figure 10:**
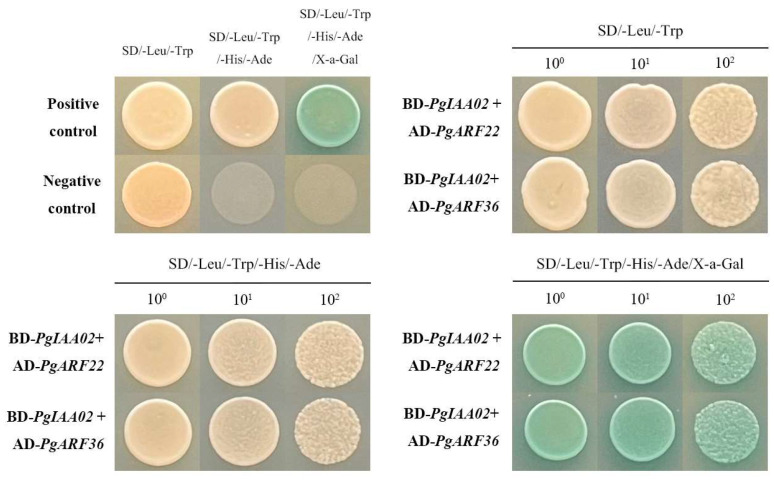
The interaction between PgIAA02 and PgARF22/36 was detected using the yeast two-hybrid system. In the system, BD (pGBKT7) and AD (pGADT7) were used as bait and prey constructs, respectively. The positive control and negative control were pGBKT7-53 + pGADT7-T and pGBKT7-Lam + pGADT7-T, respectively.

We selected *PgIAA02* and *PgIAA22/36* homologous to *AtARF719* [26]. These three genes were inserted into pGBKT7 and pGADT7 vectors to form bait proteins and prey proteins. The interaction between PgIAA02, and PgARF22 and PgARF36 was verified via Y2H detection. As shown in Figure 10, the Y2H detection results showed that PgIAA02-BD and PgARF22-AD, PgIAA02-BD, and PgARF36-AD were blue on SD/-Leu/-Trp/-His/-Ade/X-a-Gal/Aba. The interaction specificity between PgIAA02 and PgIARF22/36 was confirmed (Figure 10).

## 3. Discussion

Aux/IAA, a member of the early auxin-responsive gene family in plants, is key to auxin-mediated developmental signal transduction. It was originally isolated from *Glycine max* [27]. Aux/IAA proteins can bind to ARF transcription factors in the absence of auxin to inhibit the transcription of target genes [28]. In the presence of auxin, Aux/IAA proteins are degraded through the activity of the complex of the ubiquitin ligase protein E3 SKP 1-CULLIN 1-F-BOX (SCF) with F-BOX protein transport inhibitor response 1 (TIR 1) and its homologue, auxin signaling F-BOX 1–5 (AFB 1–5), which initiates ARF activity and leads to the transcription of genes that produce auxin responses [6].

With the rapid development of molecular biology and plant genome research techniques, many Aux/IAA proteins have also been identified and studied in different model plants, field crops, and cash crops [20,29,30,31]. However, there is a lack of analysis and functional prediction of ginseng genome-wide expression profiles. The genomic information of ginseng chromosome levels published by Wang et al. (2022) [32] provides an opportunity to systematically evaluate the function of *Aux/IAA* family genes in this plant. In this study, in total, 84 *Aux/IAA* genes were identified from the genome of *Ginseng* and combined with phylogenetic analysis and RNA-Seq data analysis; *PgIAA02* genes related to root development were screened out. Preliminary functional verification was carried out using subcellular localization, overexpression plants, and yeast two-hybrid technology, which provided clues for the further study of the biological function of *Aux/IAA* genes.

The ancestral core dicot genome of *Ginseng* underwent three rounds of WGDs (γ, Pg-β, and Pg-α) events, which led to a unique diploidization process of genome segregation and rearrangement [32]. Twenty-nine *Aux/IAA* genes were previously identified from *Arabidopsis thaliana*. Theoretically, *Panax ginseng* has approximately three times as many genes as *Arabidopsis thaliana* [33]. This suggests that it has approximately 90 *Aux/IAA* genes (Figure 1). This is corroborated by our study, which identified a total of 84 *Aux/IAA* genes from the genome of *Panax ginseng*. This suggests that the amplification of the Aux/IAA gene family in *Panax ginseng* is affected by whole-genome triplication (WGT) and fragment duplication caused by heteropoly ploidy. The *PgIAA* family genes possess different gene structures with different numbers of exons and encode Aux/IAA proteins that exhibit a wide range of size and physicochemical properties. This opens up more possibilities for this family in terms of the diversity of action plant functions (Supplementary Table S2).

Genes of different species in the same subgroup of the phylogenetic tree have high homology, so they may encode proteins with the same biological function. Many Aux/IAA proteins have been characterized in Arabidopsis and rice, so we selected *Arabidopsis thaliana*, *Oryza sativa*, and *Panax ginseng* to construct phylogenetic trees to predict the biological functions of ginseng Aux/IAA members (Figure 2) (Supplementary Table S3). For example, the anticlinal cell division that produces lateral roots at the pericycle of the protoxylem of *slr-1/iaa14* functionalized plants was inhibited, resulting in defects in lateral root formation and a decrease in the number of lateral roots [34], and the stable tissue-specific expression of SLR/IAA14 changed the development of lateral roots in *Arabidopsis* [35]. Kim et al. (2006) [36] reported that AXR2/IAA7 and AXR3/IAA17 also play an important role in root development. Therefore, we predict that *PgIAA02*, *PgIAA36*, *PgIAA69,* and *PgIAA81* in the same branch may have the same biological functions and play a role in root development.

Conservative motif and gene structure studies provided additional evidence for phylogenetic relationships. Phylogenetic analysis classified all ginseng Aux/IAA proteins into six subfamilies (Figure 3). Among them, subfamily VI is the largest subfamily in the ginseng *Aux/IAA* family genes, which accounts for 48% of all *PgIAA* genes. All members of subfamily VI lack conserved structural domain I. Domain I of the Aux/IAA proteins has been shown in Arabidopsis thaliana to be an active and portable repressor domain containing the “L × L × L” motif, which interacts with the TOPLESS (TPL) co-repressor [10]. This indicates that these PgIAA proteins have lost the ability to recruit TPL co-repressors, which are not involved in classical auxin signaling. Aux/IAA plays a stable role in transcriptional repression by binding to ARF proteins mediated by domain III and IV. Point mutations in the transcriptional negative regulators *axr3/iaa17* [37] or *axr2/iaa7* [38] caused phenotypic changes, altered domain III of these stable proteins, and impaired their ability to homodimerize and heterodimerize [39]. Conserved domain analysis showed that the PgIAA members in subgroup VI contained four complete conserved domains, which were classical Aux/IAA proteins. Therefore, it was predicted that the members of this subgroup could participate in regulating the activity of downstream ARF transcription factors through domain III and IV, thereby regulating the root development of *Ginseng*.

Gene duplication is an important mechanism for the expansion and evolution of plant gene families. The modes of gene duplication can be broadly classified into whole-genome duplication (WGD), segmental duplication (SD), tandem duplication (TD), proximal duplication (PD), transpositional duplication (TRD), and dispersal duplication (DSD), with segmental and tandem duplication considered to be the major modes of gene family expansion [40,41]. In this study, in total, 52 pairs of genes with collinearity were identified in 84 *Aux/IAA* genes, including *PgIAA02* and *PgIAA36/69/81* (Figure 5). Among them, 50 pairs of PgIAA proteins originated from segmental duplication, suggesting that recent segmental duplication events may be the main driver for the expansion of ginseng *Aux/IAA* family genes (Supplementary Table S6). Then, the evolutionary origin and homology of *Aux/IAA* genes were explored among different species. Meanwhile, six species were analyzed. Notably, five homologous genes (*PgIAA14*, *PgIAA25*, *PgIAA32*, *PgIAA68*, and *PgIAA83*) were found in *Arabidopsis thaliana* (Supplementary Figure S2). This suggests that these five genes may have been inherited from an earlier land plant ancestor. Among them, *PgIAA14* has quasi-homologous genes in *At*, *Pt*, *Dc*, *Pj*, and *Pq*, and *PgIAA32* has quasi-homologous genes in *Pt* and *Pq*, which may indicate that these two genes are more conserved during evolution.

Based on the selection pressure analysis, only 2 of the 51 pairs of *Aux/IAA* genes in *Ginseng* had a Ka/Ks value greater than one, indicating that the 2 pairs of base genes may have undergone rapid evolution after duplication and experienced positive selection pressure. It is not surprising that the Ka/Ks ratios of most ginseng *Aux/IAA* repeat gene pairs are below one, indicating that the remaining 49 pairs of ginseng *Aux/IAA* genes have experienced strong purification selection pressure during evolution and limited functional differentiation, and that their evolution is conservative (Supplementary Table S7).

The diversity of homeostatic elements can explain the diverse functions of Aux/IAA in plant development, which plays an important role in the study of gene function. We found many hormone-related cis-acting elements 1500 bp upstream of the *PgIAA* genes (Supplementary Table S5). These findings suggest that the *PgIAA* gene can enhance the response of *Ginseng* to these hormones, especially auxin, gibberellin, salicylic acid, MeJA, and abscisic acid, and regulate plant growth and development in a hormone-responsive manner (Figure 4). Cis-acting elements may be related to transcription factors located in the promoter region of the gene and participate in regulating gene expression [42].

The tissue-specific expression patterns of *Aux/IAA* genes help us to understand their functional loci in *Ginseng*. The expression patterns of 84 ginseng *Aux/IAA* genes were found to be different in 15 different tissues (Figure 6A). For example, *PgIAA05*, *PgIAA42*, and *PgIAA57* were highly expressed in three tissue sites, the periderm, cortex, and stele of the main root, but lowly expressed in the leaves. *PgIAA02*, *PgIAA36*, *PgIAA69,* and *PgIAA81* were highly expressed in the fiber roots, leg roots, and arm roots. Significant differences in the expression of *Aux/IAA* genes in the different tissue sites of *Ginseng* may be due to different modes of regulation of growth hormone-dependent transcriptional and post-transcriptional regulation and may also be related to the specificity of growth hormone perception in different tissues and the differential regulation of free growth hormone concentrations. The phylogenetic analysis showed that *PgIAA02*, *PgIAA36*, *PgIAA69,* and *PgIAA81*, which are clustered into the same branch as *AtIAA7*, *AtIAA14,* and *AtIAA17*, may play a similar role in ginseng root development [35,43,44], which is consistent with the expression pattern of *PgIAA02* and *PgIAA36* in various parts of the root, as determined via transcriptional analysis. The results of q-PCR also proved that the expression levels of *PgIAA02* and *PgIAA36* in lateral roots, fibrous roots, and adventitious roots were higher than those in other parts of the root, which strongly indicated that these genes may be related to auxin signal transduction in the development of ginseng root meristem (Figure 7). On the other hand, auxin induced the upregulation of *PgIAA02* expression in leaves. The auxin response element, the TGA element, was indeed found in the upstream promoter of PgIAA02, and the transcription factor MYB, which can bind to auxin-related elements, was found in the analysis of the transcription factor interaction network (Figure 6B). Therefore, we speculate that transcription factors can respond to auxin and regulate the transcriptional expression level of downstream *PgIAA* genes by binding to the upstream promoter of *PgIAA*.

The PgIAA02 protein is an important component of the auxin signaling pathway. Studies have shown that auxin signaling can affect the stability of the Aux/IAA protein and change the ability of the Aux/IAA protein to bind to the ARF protein, thereby regulating the expression of ARF downstream genes and participating in the biological process of root development [45]. We constructed *PgIAA02* overexpression plants and counted the root phenotype. The results showed the ability of *PgIAA02* to regulate lateral root growth and development (Figure 9). It has been reported that loss-of-function mutations in ARF7/NPH4 or ARF19 lead to a slight decrease in lateral root formation, while double arf7/arf19 mutations show a significant delay in lateral root formation, indicating that these genes can play similar functions in roots [46,47]. In yeast two-hybrid experiments, ARF7 and ARF19 proteins interact with a variety of Aux/IAA proteins. Therefore, we identified the homologous gene PgARF22/36 of ARF7/19 from the ginseng genome and performed a yeast two-hybrid experiment [48]. The potential regulatory effects of PgIAA02 on PgARF22 and PgIAA36 were further verified through Y2H experiments (Figure 10). Combined with the results of subcellular localization, we predicted that PgIAA02 could bind to PgARF22 and PgARF36 in the nucleus to change the root phenotype (Figure 8).

Based on the above analysis and results, we believe that auxin may be involved in ginseng root development through two regulatory pathways. On the one hand, auxin responds to auxin through transcription factors and affects the expression level of downstream *PgIAA* genes. On the other hand, auxin affects the stability of PgIAA proteins and initiates downstream ARF transcriptional activity through the degradation of PgIAA. During the development of ginseng roots, hormone signals and transcription factors form a complex and precise regulatory network that coordinates various life processes in plants. However, no matter which way, it is necessary for auxin to participate in the biological process of regulating ginseng root development, but the specific mechanism is still worthy of further verification and exploration.

## 4. Materials and Methods

### 4.1. Collection of Data Resources

Chromosome-level gene sequences and the latest annotation files of *Panax ginseng*, *Panax quinquefolium*, *Panax stipulesnatus*, and *Panax japonicus* were downloaded from the National Genome Sciences Data Centre (https://ngdc.cncb.ac.cn/, (accessed on 8 April 2023)) to construct local databases [32]. The amino acid sequences of Aux/IAA from *Arabidopsis thaliana* were obtained from the TAIR database (http://www.arabidopsis.org, (accessed on 8 April 2023)) [23], and the amino acid sequences of Aux/IAA from *Oryza sativa* were obtained from the NCBI database (https://www.ncbi.nlm.nih.gov/, (accessed on 8 April 2023)) [20]. *Populus trichocarpa* and *Daucus carota* genomic data were downloaded from the integrated database Phytozome v 13.0 (https://phytozome-next.jgi.doe.gov/, (accessed on 8 April 2023)).

### 4.2. Genome-Wide Identification of the Aux/IAA Family Genes in Ginseng

To fully characterize the *Aux/IAA* genes in *Ginseng*, the hidden Markov model (HMM) of the Aux/IAA structural domain (PF02309) was downloaded from the Protein Family Database (Pfam) (http://pfam.xfam.org/, (accessed on 3 June 2023)). Sequences matching the structural features were searched from the ginseng genome using HMMRER 3.2.1 software (E-value of 1 × 10^−5^). Candidate protein sequences were validated using the NCBI database (https://www.ncbi.nlm.nih.gov/cdd/, (accessed on 3 June 2023)), conserved fields (E-value of 1 × 10^−5^), and the SMART database (http://smart.embl-heidelberg.de/, (accessed on 3 June 2023)). Sequences that did not contain the PB1 structural domain were then eliminated, and the remaining genes encoding the PB1 structural domain were members of the ginseng *Aux/IAA* family genes and were used for further analysis.

### 4.3. Evolutionary Analysis of the PgIAA Family Genes

The default parameter of MAFFT (http://mafft.cbrc.jp/alignment/software/, (accessed on 3 June 2023)) was used for the multiple matching of *Ginseng Aux/IAA* family genes as well as the multiple matching of *Aux/IAAs* among other species. A *PgIAA* gene phylogenetic tree was built using the maximum likelihood IQ-TREE method based on the JTTDCMut+F+R4 model [48]. Relative branch support was assessed using 1000 bootstrap replicates. Branch length was calculated through pairwise genetic distance comparison. Missing data were processed through the pairwise deletion of gaps. The phylogenetic tree results were further annotated using iTOL (https://itol.embl.de/, (accessed on 3 June 2023)).

### 4.4. PgIAA Gene Structure and Protein-Conserved Sequence Analysis

The *PgIAA* genes were mapped on chromosomes using MapChart 2.0 software and renamed according to the chromosomal location of the *PgIAA* genes. The biochemical properties of PgIAA proteins were analyzed using the ProtParam Expasy online software tool (https://web.expasy.org/protparam/ (accessed on 22 July 2023)), and their molecular weights, isoelectric points, and hydrophilic properties were obtained. Gene structure information on the exon and intron coordinates of PgIAA genes was obtained and analyzed from *Panax ginseng* genome annotation files. The conserved protein motifs of PgIAA were identified using MEME local software (version 4.12.0) in Linux.

### 4.5. Analysis of Cis-Regulatory Elements in the PgIAA Promoter

The 1500 bp genomic sequence upstream of the transcription start site (TSS) of the *PgIAA* genes was extracted from the ginseng genomic data resource. Meanwhile, the PlantCARE website analyzed the cis-acting elements in the promoter region. (http://bioinformatics.psb.ugent.be/webtools/plantcare/html/ (accessed on 22 July 2023)).

### 4.6. Chromosome Duplication, Collinearity, and Evolutionary Analysis

Interspecific and intraspecific collinearity analyses of proteins were performed using the MCScanX program to further elucidate and characterize orthologous and paralogous homologous genes in the replication events of the *Aux/IAA* family genes in *Ginseng*. KaKs_Calculator 2.0 was used to calculate the non-synonymous substitution rate (Ka) and the synonymous substitution rate (Ks) of the replicated gene pairs and to analyze the environmental selection pressure through the Ka/Ks ratio.

### 4.7. PgIAA Genes Expression Analysis

To distinguish the expression pattern of *PgIAA* genes in different organs and tissue sites, RNA-seq data were firstly acquired from the NCBI SRA database (accession numbers PRJNA302556 and PRJNA369187) [26]. They were preprocessed and normalized to obtain TPM values. In addition, the heat mapping of *PgIAA* genes was performed using Evolview 2.0 software (https://evolgenius.info/, (accessed on 22 July 2023)).

### 4.8. Real-Time Quantitative qRT-PCR Analysis

Total RNA was extracted from each sample using RNAprep pure Plant Kit (TIANGEN) and reverse-transcribed into cDNA, with the final cDNA product diluted 10 times before use. The primer design is shown in Supplementary Table S1. Amplification reactions were performed on a LightCycler 96 (ROCHE) using PerfectStart^®^ qPCR SuperMix (Transgen). The qRT-PCR assay normalized the expression levels of the *PgIAA* gene in different samples with the β-actin gene as a control (3 independent biological replicates and 3 technical replicates). Relative expression levels were analyzed using the standard curve method described above [49].

### 4.9. Subcellular Localization

The CDS sequences of PgIAA02/42/58 were cloned from Panax ginseng cDNA using PgIAA02-F/R, PgIAA42-F/R, and PgIAA58-F/R primers (Supplementary Table S1). The pCAMBIA2300-GFP vector was digested with BamHI, and the amplification product was inserted into the linearized pCAMBIA2300-GFP vector using pEASY^®^-Basic Seamless Cloning and Assembly Kit, which was verified via DNA sequencing. The 4 vectors were converted into Agrobacterium GV3101. As described earlier, the Agrobacterium cultures of each structure were re-suspended and mixed, and then they infiltrated Nicotiana tabacum leaves.

### 4.10. Identification of the Root Phenotypes of Arabidopsis with PgIAA02 Overexpression

The *PgIAA02* sequence was inserted into the pCAMBIA1300 vector, and the correctly sequenced plasmid was transformed into Agrobacterium tumefaciens EHA105, coated on LB medium containing antibiotics, and incubated in the dark at 28 °C for 2 days. Agrobacterium tumefaciens was resuspended in 5% sucrose solution (OD = 0.8), and wild-type Arabidopsis thaliana plants were transformed using the floral maceration method [50]. After 2 weeks of germination of transgenic seeds on MS + thaumatin plates, the positive plants were transferred to soil for further cultivation. The leaves of the positive plants were taken for genomic DNA extraction, and the transgenic lines were identified via PCR using the primers (HPT-F and HPT-R) shown in Supplementary Table S1.

### 4.11. Statistics of Root Phenotypes

*Arabidopsis thaliana* lateral root lengths were measured using a body microscope, and the data were statistically analyzed using Graphpad prism V8.0 software. Significance of differences (*p* < 0.05) was tested via one-way ANOVA, and data significance was determined through Waller–Duncan letter labeling (*p* < 0.05).

### 4.12. Yeast Two-Hybrid Assay

The *PgIAA02* sequence was inserted into the pGADT7 vector, and the *PgARF22* and *PgARF36* sequences were inserted into the pGBKT7 vector to generate prey and bait vectors (Supplementary Table S1) (positive control: pGBKT7-53 + pGADT7-T; negative control: pGBKT7-Lam + pGADT7-T; experimental groups: BD-PgARF22 + AD-PgIAA02 and BD-PgARF36 + AD-PgIAA02). Each of the above four combinations was co-transformed into the Y2H Gold yeast strain, which was incubated on DDO (SD/-Leu/-Trp) plates at 30 °C for 4 days in the dark to identify positive strains (200 ng·mL^−1^). The self-activation of each exogenous protein and the reciprocal effect of reciprocal combinations were confirmed on QDO (SD/-Leu/-Trp/-His/-Ade) plates and QDO/X-a-Gal/Aba plates.

## 5. Conclusions

In total, 84 ginseng *Aux/IAA* gene family members were identified in this study. Phylogenetic analysis showed that the *PgIAA* genes could be divided into six subgroups. By analyzing the structure, evolutionary history, cis-elements, transcription factor regulatory network, transcription analysis, and q-PCR verification of *PgIAA* genes, the gene *PgIAA02*, which may be involved in the regulation of root development, was screened out. The function of PgIAA02 in root development was preliminarily verified through subcellular localization, overexpression plants, and a yeast two-hybrid assay. These findings provide a valuable theoretical basis for studying the role of Aux/IAA in the regulation mechanism of ginseng root development and provide valuable clues for the further study of the ginseng auxin signal transduction pathway and auxin regulation mechanism.

## Data Availability

Data are contained within the article and Supplementary Materials.

## Supplementary Materials

The following supporting information can be downloaded at https://www.mdpi.com/article/10.3390/ijms25063470/s1.

## References

[1] Thimann K.V., Went F.W. (1934). On the chemical nature of the root forming hormone. Proc. K. Ned. Akad. Van Wet..

[2] Knox K., Grierson C.S., Leyser O.J.D. (2003). AXR3 and SHY2 interact to regulate root hair development. Development.

[3] Benjamins R., Scheres B. (2008). Auxin: The Looping Star in Plant Development. Annu. Rev. Plant Biol..

[4] Shin R., Burch A.Y., Huppert K.A., Tiwari S.B., Murphy A.S., Guilfoyle T.J., Schachtman D.P. (2007). The Arabidopsis Transcription Factor MYB77 Modulates Auxin Signal Transduction. Plant Cell.

[5] Yu C.L., Sun C.D., Shen C.J., Wang S.K., Liu F., Liu Y., Chen Y.L., Li C.Y., Qian Q., Aryal B. (2015). The auxin transporter, OsAUX1, is involved in primary root and root hair elongation and in Cd stress responses in rice (*Oryzasativa* L.). Plant J..

[6] Figueiredo M.R.A., Strader L.C. (2022). Intrinsic and extrinsic regulators of Aux/IAA protein degradation dynamics. Trends Biochem. Sci..

[7] Guilfoyle T.J., Hagen G. (2007). Auxin response factors. Curr. Opin. Plant Biol..

[8] Ren H., Gray W.M. (2015). SAUR Proteins as Effectors of Hormonal and Environmental Signals in Plant Growth. Mol. Plant.

[9] Wojtaczka P., Ciarkowska A., Starzynska E., Ostrowski M. (2022). The GH3 amidosynthetases family and their role in metabolic crosstalk modulation of plant signaling compounds. Phytochemistry.

[10] Szemenyei H., Hannon M., Long J.A. (2008). TOPLESS mediates auxin-dependent transcriptional repression during Arabidopsis embryogenesis. Science.

[11] Calderón Villalobos L.I., Lee S., De Oliveira C., Ivetac A., Brandt W., Armitage L., Sheard L.B., Tan X., Parry G., Mao H. (2012). A combinatorial TIR1/AFB-Aux/IAA co-receptor system for differential sensing of auxin. Nat. Chem. Biol..

[12] Leyser H.M., Lincoln C.A., Timpte C., Lammer D., Turner J., Estelle M. (1993). Arabidopsis auxin-resistance gene AXR1 encodes a protein related to ubiquitin-activating enzyme E1. Nature.

[13] Moss B.L., Mao H., Guseman J.M., Hinds T.R., Hellmuth A., Kovenock M., Noorassa A., Lanctot A., Villalobos L.I.A.C., Zheng N. (2015). Rate Motifs Tune Auxin/Indole-3-Acetic Acid Degradation Dynamics. Plant Physiol..

[14] Arase F., Nishitani H., Egusa M., Nishimoto N., Sakurai S., Sakamoto N., Kaminaka H. (2012). IAA8 involved in lateral root formation interacts with the TIR1 auxin receptor and ARF transcription factors in Arabidopsis. PLoS ONE.

[15] Guseman J.M., Hellmuth A., Lanctot A., Feldman T.P., Moss B.L., Klavins E., Villalobos L.I.A.C., Nemhauser J.L. (2015). Auxin-induced degradation dynamics set the pace for lateral root development. Development.

[16] Notaguchi M., Wolf S., Lucas W.J. (2012). Phloem-mobile Aux/IAA transcripts target to the root tip and modify root architecture. J. Integr. Plant Biol..

[17] Jun N., Gaohang W., Zhenxing Z., Huanhuan Z., Yunrong W., Ping W. (2011). OsIAA23-mediated auxin signaling defines postembryonic maintenance of QC in rice. Plant J..

[18] Hamann T., Benkova E., Bäurle I., Kientz M., Jürgens G. (2002). The Arabidopsis BODENLOS gene encodes an auxin response protein inhibiting MONOPTEROS-mediated embryo patterning. Genes Dev..

[19] Tatematsu K., Kumagai S., Muto H., Sato A., Watahiki M.K., Harper R.M., Liscum E., Yamamoto K.T. (2004). MASSUGU2 encodes Aux/IAA19, an auxin-regulated protein that functions together with the transcriptional activator NPH4/ARF7 to regulate differential growth responses of hypocotyl and formation of lateral roots in Arabidopsis thaliana. Plant Cell.

[20] Jain M., Kaur N., Garg R., Thakur J.K., Tyagi A.K., Khurana J.P. (2006). Structure and expression analysis of early auxin-responsive Aux/IAA gene family in rice (*Oryza sativa*). Funct. Integr. Genom..

[21] Liu K., Yuan C., Feng S., Zhong S., Li H., Zhong J., Shen C., Liu J. (2017). Genome-wide analysis and characterization of Aux/IAA family genes related to fruit ripening in papaya (*Carica papaya* L.). BMC Genom..

[22] Paul P., Dhandapani V., Rameneni J.J., Li X., Sivanandhan G., Choi S.R., Pang W., Im S., Lim Y.P. (2016). Genome-Wide Analysis and Characterization of Aux/IAA Family Genes in *Brassica rapa*. PLoS ONE.

[23] Remington D.L., Vision T.J., Guilfoyle T.J., Reed J.W. (2004). Contrasting modes of diversification in the Aux/IAA and ARF gene families. Plant Physiol..

[24] Wang K., Jiang S., Sun C., Lin Y., Yin R., Wang Y., Zhang M. (2015). The Spatial and Temporal Transcriptomic Landscapes of Ginseng, *Panax ginseng* C. A. Meyer. Sci. Rep..

[25] Zhang J., Su H., Zhang L., Liao B.-S., Xiao S.-M., Dong L.-L., Hu Z.-G., Wang P., Li X.-W., Huang Z.-H. (2017). Comprehensive Characterization for Ginsenosides Biosynthesis in Ginseng Root by Integration Analysis of Chemical and Transcriptome. Molecules.

[26] Yan M., Yan Y., Wang P., Wang Y., Piao X., Di P., Yang D.-C. (2023). Genome-Wide Identification and Expression Analysis of Auxin Response Factor (ARF) Gene Family in Panax ginseng Indicates Its Possible Roles in Root Development. Plants.

[27] Walker J.C., Key J.L. (1982). Isolation of cloned cDNAs to auxin-responsive poly(A)RNAs of elongating soybean hypocotyl. Proc. Natl. Acad. Sci. USA.

[28] Luo J., Zhou J.J., Zhang J.Z. (2018). Aux/IAA Gene Family in Plants: Molecular Structure, Regulation, and Function. Int. J. Mol. Sci..

[29] Li H., Wang B., Zhang Q., Wang J., King G.J., Liu K. (2017). Genome-wide analysis of the auxin/indoleacetic acid (Aux/IAA) gene family in allotetraploid rapeseed (*Brassica napus* L.). BMC Plant Biol..

[30] Liscum E., Reed J.W. (2002). Genetics of Aux/IAA and ARF action in plant growth and development. Plant Mol. Biol..

[31] Wu W., Liu Y., Wang Y., Li H., Liu J., Tan J., He J., Bai J., Ma H. (2017). Evolution Analysis of the Aux/IAA Gene Family in Plants Shows Dual Origins and Variable Nuclear Localization Signals. Int. J. Mol. Sci..

[32] Wang Z.H., Wang X.-F., Lu T., Li M.-R., Jiang P., Zhao J., Liu S.-T., Fu X.-Q., Wendel J.F., Van de Peer Y. (2022). Reshuffling of the ancestral core-eudicot genome shaped chromatin topology and epigenetic modification in Panax. Nat. Commun..

[33] Park J.Y., Koo D.H., Hong C.P., Jeon J.W., Lee S.H., Yun P.Y., Park B.S., Kim H.R., Bang J.W., Plaha P. (2005). Physical mapping and microsynteny of *Brassica rapa* ssp. *pekinensis* genome corresponding to a 222 kbp gene-rich region of *Arabidopsis chromosome* 4 and partially duplicated on chromosome 5. Mol. Genet. Genom..

[34] Fukaki H., Tameda S., Masuda H., Tasaka M. (2002). Lateral root formation is blocked by a gain-of-function mutation in the SOLITARY-ROOT/IAA14 gene of *Arabidopsis*. Plant J..

[35] Fukaki H., Nakao Y., Okushima Y., Theologis A., Tasaka M. (2005). Tissue-specific expression of stabilized SOLITARY-ROOT/IAA14 alters lateral root development in *Arabidopsis*. Plant J..

[36] Kim H., Park P., Hwang H., Lee S., Oh M., Kim S. (2006). Brassinosteroid signals control expression of the AXR3/IAA17 gene in the cross-talk point with auxin in root development. Biosci. Biotechnol. Biochem..

[37] Rouse D., Mackay P., Stirnberg P., Estelle M., Leyser O. (1998). Changes in auxin response from mutations in an AUX/IAA gene. Science.

[38] Nagpal P., Walker L.M., Young J.C., Sonawala A., Timpte C., Estelle M., Reed J.W. (2000). AXR2 encodes a member of the Aux/IAA protein family. Plant Physiol..

[39] Ouellet F., Overvoorde P.J., Theologis A. (2001). IAA17/AXR3: Biochemical insight into an auxin mutant phenotype. Plant Cell.

[40] Cannon S.B., Mitra A., Baumgarten A., Young N.D., May G. (2004). The roles of segmental and tandem gene duplication in the evolution of large gene families in *Arabidopsis thaliana*. BMC Plant Biol..

[41] Qiao X., Li Q., Yin H., Qi K., Li L., Wang R., Zhang S., Paterson A.H. (2019). Gene duplication and evolution in recurring polyploidization-diploidization cycles in plants. Genome Biol..

[42] Yin J., Hou L., Jiang X., Yang J., He Y., Zhou X., Zhu X., Gong A., Zhu Y., Chen Z. (2021). Identification and validation of reference genes for quantitative real-time PCR studies in alligatorweed (*Alternanthera philoxeroides*). Weed Sci..

[43] Leyser H.M., Pickett F.B., Dharmasiri S., Estelle M. (1996). Mutations in the AXR3 gene of Arabidopsis result in altered auxin response including ectopic expression from the SAUR-AC1 promoter. Plant J..

[44] Wilson A.K., Pickett F.B., Turner J.C., Estelle M. (1990). A dominant mutation in *Arabidopsis* confers resistance to auxin, ethylene and abscisic acid. Mol. Gen. Genet..

[45] Peng Y., Jiang S., Wang J., Xu X., Gong X., Jin W., Song C., Dong Z., Sun S., Li Y. (2023). Control of lateral root initiation by DA3 in Arabidopsis. Cell Rep..

[46] Wilmoth J.C., Wang S., Tiwari S.B., Joshi A.D., Hagen G., Guilfoyle T.J., Alonso J.M., Ecker J.R., Reed J.W. (2005). NPH4/ARF7 and ARF19 promote leaf expansion and auxin-induced lateral root formation. Plant J..

[47] Okushima Y., Overvoorde P.J., Arima K., Alonso J.M., Chan A., Chang C., Ecker J.R., Hughes B., Lui A., Nguyen D. (2005). Functional genomic analysis of the AUXIN RESPONSE FACTOR gene family members in Arabidopsis thaliana: Unique and overlapping functions of ARF7 and ARF19. Plant Cell.

[48] Di P., Wang P., Yan M., Han P., Huang X., Yin L., Yan Y., Xu Y., Wang Y. (2021). Genome-wide characterization and analysis of WRKY transcription factors in Panax ginseng. BMC Genom..

[49] Ma H., Zhao J. (2010). Genome-wide identification, classification, and expression analysis of the arabinogalactan protein gene family in rice (*Oryza sativa* L.). J. Exp. Bot..

[50] Clough S.J., Bent A.F. (1998). Floral dip: A simplified method for Agrobacterium-mediated transformation of *Arabidopsis thaliana*. Plant J..

