# Supplementary material for: Genome-Wide Identification and Analysis of the Aux/IAA Gene Family in Panax ginseng: Evidence for the Role of PgIAA02 in Lateral Root Development
Source: Int J Mol Sci. 2024 Mar 19;25(6):3470. doi: 10.3390/ijms25063470 (PMC10971203; doi:10.3390/ijms25063470)
Supplement: Supplementary file 1 [file ijms-25-03470-s001.zip › Supplementary Figure S2.pdf]

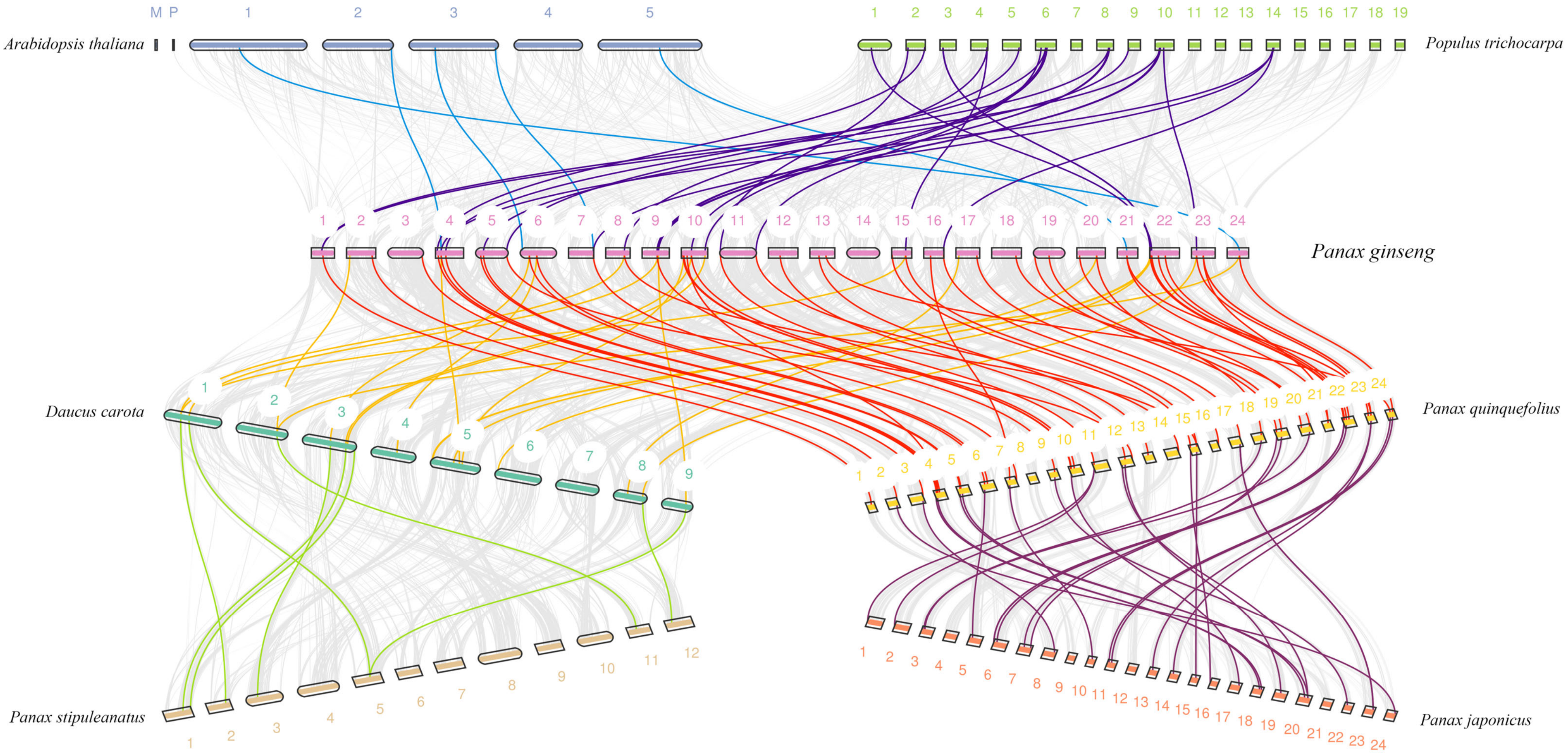

Supplementary Figure S2. Co-linear analysis of *Panax ginseng*, *Panax quinquefolius*, *Panax stipulesnatus*, *Panax japonicus*, *Arabidopsis thaliana*, *Populus trichocarpa* and *Daucus carota* genes. Grey lines in the background represent blocks of colinearity between species, and different coloured lines highlight Aux/IAA gene pairs with colinearity.
