# Supplementary figures and images for: Genome-Wide Identification and Analysis of the Aux/IAA Gene Family in Panax ginseng: Evidence for the Role of PgIAA02 in Lateral Root Development
Source: Int J Mol Sci. 2024 Mar 19;25(6):3470. doi: 10.3390/ijms25063470 (PMC10971203; doi:10.3390/ijms25063470)

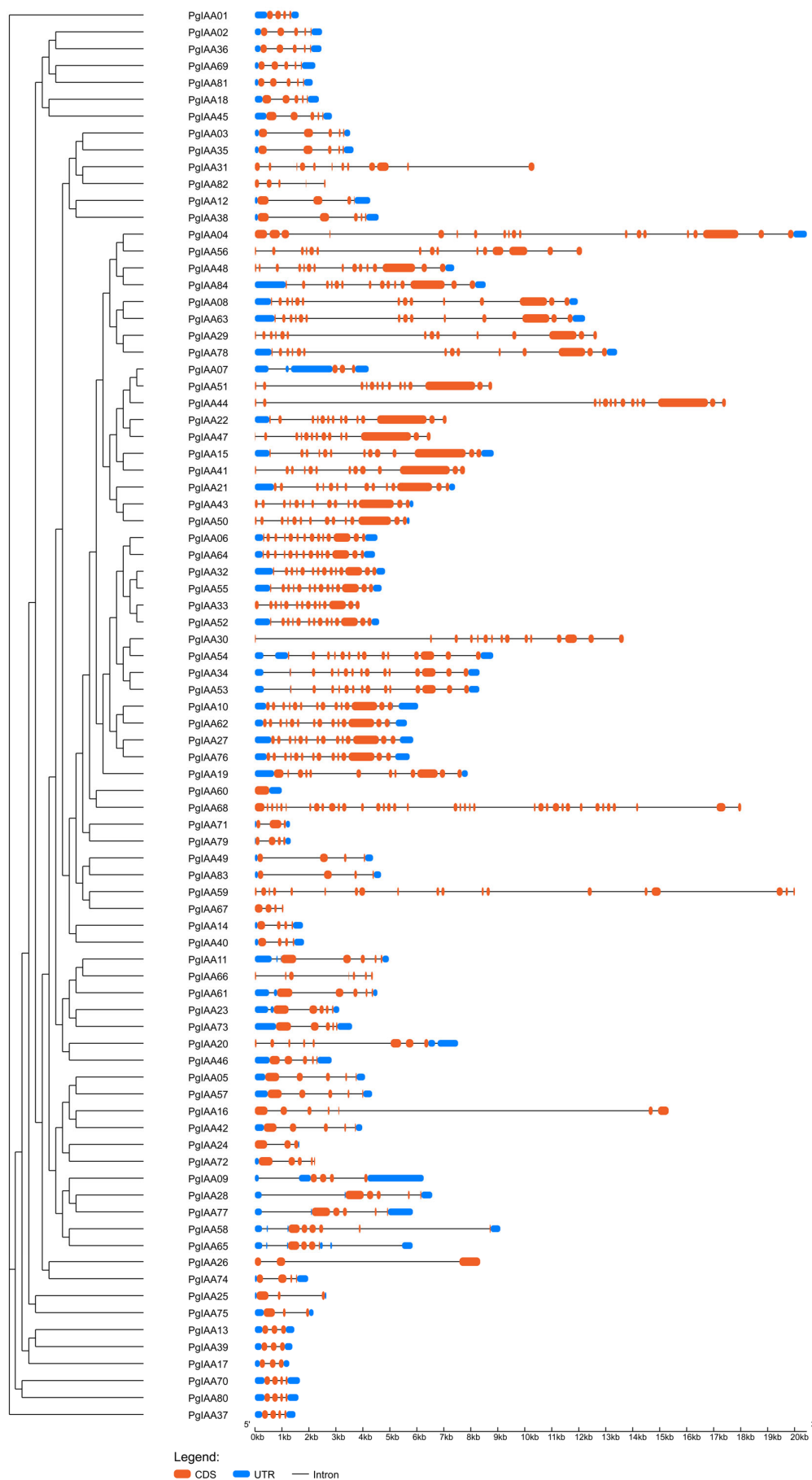

**Supplementary Figure S1.** Phylogenetic tree and gene structure of 84 PgIAA genes.

Supplement: Supplementary file 1 [file ijms-25-03470-s001.zip › Supplementary Figure S1.pdf]
